# Changes in demographic and neurological characteristics in patients of a tertiary spinal cord injury centre with acquired traumatic or non-traumatic spinal cord injury/disease over a 25-year period. Implications for bladder management

**DOI:** 10.1038/s41394-026-00741-5

**Published:** 2026-08-29

**Authors:** Ralf Böthig, Ines Kurze, Birgitt Kowald, Roland Thietje, Peter Wolna, Klaus Golka

**Affiliations:** 1 Department of Neuro-Urology, Centre for Spinal Cord Injuries, BG Klinikum Hamburg, Hamburg, Germany; 2 Centre for Expert Opinions, BG Klinikum Hamburg, Hamburg, Germany; 3 Department of Paraplegiology and Neuro-Urology, Centre for Spinal Cord Injuries, Zentralklinik Bad Berka, Bad Berka, Germany; 4 Centre for Clinical Research, BG Klinikum Hamburg, Hamburg, Germany; 5 Centre for Spinal Cord Injuries, BG Klinikum Hamburg, Hamburg, Germany; 6 Ginsterweg 14, Halle, Germany; 7 Leibniz Research Centre for Working Environment and Human Factors at TU Dortmund (IfADo), Dortmund, Germany

**Keywords:** Rehabilitation, Quality of life

## Abstract

**Study design:**

Retrospective data analysis.

**Objective:**

To investigate the changes in demographic and neurological characteristics in patients with acquired traumatic or non-traumatic spinal cord injury/disease (SCI/D) over a 25-year period and their implications for rehabilitation and bladder management.

**Setting:**

Specialized tertiary German center for spinal cord injury.

**Methods:**

We retrospectively evaluated the data of SCI/D patients admitted to initial rehabilitation between January 1995 and December 1999 (1st period, n = 461) and between January 2020 and December 2021 (2nd period, n = 441).

**Results:**

In the period from January 2020 to December 2021, SCI/D patients were significantly older at the onset of paralysis and more often female, paralyzed due to a non-traumatic cause or incompletely paralyzed than 25 years ago. The proportion of young adult patients (16–45-year-olds) fell from 61.4 to 23.1%, while the proportion of the over 61-year-olds rose from 14.2 to 49.3%. The proportion of non-traumatic SCI/D rose from 20.8 to 53.1%. The duration of primary rehabilitation decreased significantly from 158 to 85 days. The proportion of SCI/D patients with intermittent catheterisation on discharge from primary rehabilitation decreased from 62.7 to 27.2%, while the proportion of patients with indwelling catheter at the time of discharge from primary rehabilitation increased from 10.8 to 52.4%.

**Conclusion:**

The proven demographic changes should lead to considerations about targeted objectives and quality criteria for initial rehabilitation after acquired SCI/D. Further research on this topic should include patient-reported outcome measures. Adequate bladder management of these often elderly patients with non-traumatic SCI/D remains a challenge.

## Introduction

Spinal cord injury/disease (SCI/D) is a devastating condition that results in neurological impairment of motor, sensory and autonomic function and represents a significant burden on those affected, their families and also on the healthcare system [1]. People with SCI/D require highly specialised multidisciplinary and multiprofessional services, which lead to considerable costs in the healthcare system in the rehabilitation and in the long term, but above all in the acute phase after the onset of SCI/D [2]. It is therefore important for healthcare professionals and policy makers to have valid information on changes over time in this population.

In the context of demographic and social changes, age, ratio of genders, neurological paralysis characteristics and the comorbidities of patients admitted to a spinal cord injury centre for initial rehabilitation after SCI/D have changed considerably in recent decades [3, 4]. The causes of SCI/D and the ratio between traumatic spinal cord injury (TSCI) and non-traumatic SCI (NTSCI) are also subject to long-term changes. These facts are observed and reflected in many established spinal cord injury centres worldwide, but profound scientific studies that would prove these changes are still rare [5]. Although up-to-date data are crucial for the planning and delivery of health services, there is surprisingly little data on TSCI and even less on NTSCI and their changes over time [6].

In recent years, however, scientific interest in these issues has increased significantly. Several studies have recently been published on the epidemiological characteristics of TSCI from a global perspective [7] or from China [8–10], South Korea [4], the Netherlands [11], the USA [12, 13], Japan [14], or Canada [15]. There are also a few epidemiological studies on NTSCI, e.g., from South Korea [3, 16] or Canada [17]. Only very few studies deal explicitly with epidemiological and neurological differences [6, 18] or their changes over the last decades in both TSCI and NTSCI [5].

For Germany, there is only information available on the nationwide SCI/D incidence from the Federal Bureau of Statistics for the period from 2013 to 2020, based on the evaluation of ICD-10 codes [19], but data on the ASIA classification, for example, was not recorded. Therefore, only limited conclusions can be drawn from this health insurance-based data regarding the neurological characteristics of SCI/D and their changes over time. However, these changes are likely to have a significant impact, including and especially on the possibilities for adequate bladder and bowel management in SCI/D patients, which are crucial for the life expectancy and quality of life of those affected. However, the influence of the changes in demographic and neurological characteristics in SCI/D patients over recent years on the choice of bladder management at the end of initial rehabilitation has not yet been thoroughly investigated. To add relevant data from a large tertiary spinal cord injury centre in Germany to broaden the general database is the aim of this study.

## Methods

The electronical patient database of the Center for Spinal Cord Injuries at BG Klinikum Hamburg, a tertiary spinal injury center, was retrospectively searched to identify patients with acquired SCI/D admitted to initial rehabilitation between January 1995 and December 1999 (1st period, n = 461) und between January 2020 and December 2021 (2nd period, n = 441).

Demographic, neurological-paraplegiological (e.g., level and severity of SCI/D) and neuro-urological data (type of bladder management) as well as data on the cause of SCI/D (traumatic or non-traumatic), duration of initial treatment and discharge management of SCI/D patients were recorded.

The extracted data were entered into a database and anonymized upon entry.

The neurological level of SCI/D was classified according to the International Standards for Neurological Classification of SCI (ISNCSCI). The severity (degree of impairment) was classified in accordance with the American Spinal Injury Association (ASIA) Impairment Scale (AIS) [20]. Neurological level of injury and severity of the SCI/D were categorised as follows: C1–4 AIS A, B and C; C5–8 AIS A, B and C; T1–S3 AIS A, B and C; AIS D at any injury level [21].

The results are described in accordance with the standardised recommendations of DeVivo et al. [22].

First, the data collected was summarised via descriptive statistics. In addition, exploratory p-values were calculated. The significance level was set to 0.05. Frequencies were statistically analysed using the Chi-Square Test or, if the sample size was too small, Fisher’s Exact Test was applied. The Wilcoxon Rank Sum Test was used to compare numerical data. Second, R’s glm() function of the free software R (version 4.5.2, R foundation, 2025) was used to perform logistic regression analysis. Bladder management by transurethral indwelling catheterisation or suprapubic indwelling catheterisation was modelled with factors Gender, Age, SCl/D cause and Severity of injury. Descriptive statistical analyses were performed using SAS 9.4 software (SAS Institute Inc., Cary, NC, USA).

All applicable institutional and governmental regulations concerning the ethical use of the data were observed. The ethics committee of the University of Lübeck (AZ 2023-747) approved the study.

The elaboration of the study followed the recommendations of the STROBE statement.

## Results

Table 1 shows a comparison of the demographic and neurological data in the two data collection periods 1995–1999 (n = 461) and 2020–2021 (n = 441). Significant changes were found in the main categories “gender”, “age”, the ratio between TSCI and NTSCI, as well as in all subcategories of ”severity of injury”.

**Table 1:** Demographic and neurological characteristics in periods 1 and 2.

	1995–1999		2020–2021		p-value
	N	%	N	%	
All	461		441		
Gender					**0.0110**
Male	339	73.5	290	65.8	
Female	122	26.5	151	34.2	
Age					**<0.0001**
0–15	11	2.4	1	0.2	
16–30	163	35.4	49	11.1	
31–45	120	26.0	53	12.0	
46–60	102	22.1	121	27.4	
61–75	56	12.2	133	30.2	
76+	9	2.0	84	19.1	
Spinal cord injury					**<0.0001**
Traumatic (TSCI)	365	79.2	207	46.9	
Non-traumatic (NTSCI)	96	20.8	234	53.1	
Severity of injury					**<0.0001**
C1-C4, AIS A, B, C	76	16.5	59	13.4	
C5-C8, AIS A, B, C	75	16.3	32	7.3	
T1-S5, AIS A, B, C	182	39.5	169	38.3	
All AIS D	128	27.8	181	41.0	
Tetra compl. (C1-C8, AIS A)	85	39.4	36	22.0	**0.0003**
Tetra incompl. (C1-C8, AIS B, C, D)	131	60.6	128	78.1	
Para compl. (T1-S5 AIS A)	127	51.8	70	25.3	**<0.0001**
Para incompl. (T1-S5, AIS B, C, D)	118	48.2	207	74.7	

*compl*. complete; *incompl*. incomplete.

The most impressive changes over time are the shift in the age of primary rehabilitation patients and in the proportion of traumatic (TSCI) and non-traumatic (NTSCI) causes of SCI/D. For example, the proportion of young adult patients (16–45-year-olds) fell from 61.4 to 23.1%, while the proportion of older patients (over 61-year-olds) rose from 14.2 to 49.3%. The proportion of NTSCI rose from 20.8 to 53.1%.

The proportion of NTSCI patients (Table 2) increased significantly during the second study period (2020–2021) compared to the first study period (1995–1999) for all patients (from 20.8 to 53.1%), for male patients (from 14.8 to 48.3%) and also for female patients (from 37.7 to 62.3%). In terms of injury severity, there was also a significant increase in the proportion of NTSCI cases in all categories, but particularly among incomplete paraplegics (from 37.7 to 72.5%).

**Table 2:** Changes in the ratio of traumatic spinal cord injury to non-traumatic spinal cord injury in periods 1 and 2.

	1995–1999					2020–2021					p-value
	All	TSCI		NTSCI		All	TSCI		NTSCI		
	n	n	%	n	%	n	N	%	n	%	
All	461	365	79.2	96	20.8	441	207	46.9	234	53.1	**<0.0001**
Male	339	289	85.2	50	14.8	290	150	51.7	140	48.3	**<0.0001**
Female	122	76	62.3	46	37.7	151	57	37.7	94	62.3	**<0.0001**
Tetra compl.	85	82	96.5	3	3.5	36	30	83.3	6	16.7	**0,0118**
Tetra incompl.	131	106	80.9	25	19.1	128	83	64.8	45	35.2	**0.0036**
Para compl.	127	103	81.1	24	18.9	70	37	52.9	33	47.1	**<0.0001**
Para incompl.	118	74	62.7	44	37.3	207	57	27.5	150	72.5	**<0.0001**

*TSCI* traumatic spinal cord injury, *NTSCI* non-traumatic spinal cord injury, *compl*. complete, *incompl*. incomplete.

Table 3 shows the changes in discharge management and bladder management over time. Here, too, significant changes can be found in all main categories. The proportion of SCI/D patients who emptied the bladder by intermittent catheterisation (IC) decreased from 62.7 to 27.2%, while the proportion of patients with indwelling catheter drainage of the bladder at the time of discharge from primary rehabilitation increased from 10.8 to 52.4%.

**Table 3:** Discharge destination and bladder management at discharge in periods 1 and 2.

	1995–1999		2020–2021		p-value
	N	%	N	%	
Discharge destination					**<0.0001**
Nursing home	32	6.9	85	19.3	
Hospital	13	2.8	10	2.3	
Rehab facility	17	3.7	3	0.7	
Previous place of residence	257	55.8	297	67.4	
New, disabled-friendly converted home	131	28.4	26	5.9	
Other	3	0.6	2	0.4	
Bladder management					**<0.0001**
IC (AIC + ISC)	289	62,7	120	27.2	
AIC	97	21.0	2	0.4	
ISC	192	41.6	118	26.8	
IndC (TUC + SPC)	50	10.8	231	52.4	
TUC	6	1.3	83	18.8	
SPC	44	9.6	148	33.6	
SPC (during initial rehab.)	22	4.8	115	26.1	
SPC (prior to initial rehab.)	22	4.8	33	7.5	
Reflex voiding	20	4.3	17	3.8	
Voluntary voiding	102	22.1	73	16.6	

*IC* intermittent catheterisation, *AIC* assisted intermittent catheterisation, *ISC* intermittent self-catheterisation, *IndC* indwelling catheter, *TUC* transurethral indwelling catheterisation, *SPC* suprapubic indwelling catheterisation.

The duration of primary rehabilitation after acquired SCI/D decreased significantly in all categories between 1995–1999 and 2020–2021 (Table 4). For example, the primary rehabilitation period for 46–60-year-olds has halved from 188 to 84 days. The initial rehabilitation period for tetraplegics has halved from 244 to 122 days. The length of primary rehabilitation was significantly shorter for patients with NTSCI than for patients with TSCI in both time periods.

**Table 4:** Median inpatient length of stay (in days) in initial rehabilitation in periods 1 and 2.

	1995–1999		2020–2021		p-value*
	n	Median (IQR)	n	Median (IQR)	
All	461	158.0 (105.0, 232.0)	441	85.0 (55.5, 397.0)	**<0.0001**
Gender					
Male	339	169.0 (109.0, 238.0)	290	89.0 (55.0, 128.0)	**<0.0001**
Female	122	141.0 (98.0, 210.0)	151	80.0 (56.0, 109.0)	**<0.0001**
Age					
0–15	11	151.0 (101.0, 210.0)	1	17.0 (17.0, 17.0)	n/a
16–30	163	168.0 (102.0, 246.0)	49	96.5 (60.0, 141.5)	**<0.0001**
31–45	120	156.0 (109.5, 226.0	53	85.0 (51.0, 133.0)	**<0.0001**
46–60	102	188.0 (112.0, 265.0)	121	84.0 (53.0, 121.0)	**<0.0001**
61–75	56	140.5 (87.0, 187.5)	133	96.0 (64.0, 129.0)	**0.0003**
76+	9	119.0 (95.0, 137.0)	84	68.5 (43.5, 89.0)	**0.0009**
Spinal cord injury					
Traumatic	365	173.0 (116.0, 241.0)	207	101.0 (65.0, 144.0)	**<0.0001**
Non-traumatic	96	122.0 (82.5, 183.5)	234	74.5 (44.0, 98.0)	**<0.0001**
Severity					
C1-C4, AIS A, B, C	76	243.5 (179.5, 319.5)	59	122.0 (76.0, 157.0)	**<0.0001**
C5-C8, AIS A, B, C	75	236.0 (153.0, 294.0)	32	126.0 (89.0, 153.5)	**<0.0001**
T1-S5, AIS A, B, C	182	142.0 (111.0, 188.0)	169	91.0 (63.0, 112.0)	**<0.0001**
All AIS D	128	111.0 (65.0, 175.5)	181	70.0 (43.0, 94.0)	**<0.0001**

^*^Wilcoxon rank-sum test (two-sided);

*n/a* not applicable due to small number.

The average age of patients with acquired SCI/D increased by 18.7 years between the data collection periods (Table 5). This significant increase in age can be observed in all categories examined, most markedly in the neurological severity category C5-C8 AIS A, B, C. A smaller increase in age was only seen in SCI/D patients with permanent catheter drainage of the urinary bladder, because these patients were also older in the first data collection period than patients with other bladder management.

**Table 5:** Median age in periods 1 and 2, stratified for different subcategories.

	1995–1999		2020–2021		p-value*
	n	Median (IQR)	n	Median (IQR)	
All	461	36.0 (24.0, 54.0)	441	60.0 (48.0, 72.0)	**<0.0001**
Gender					
Male	339	34.0 (24.0, 52.0)	290	58.5 (43.0, 70.0)	**<0.0001**
Female	122	46.0 (29.0, 58.0)	151	65.0 (55.0, 76.0)	**<0.0001**
Spinal cord injury					
Traumatic	365	33.0 (23.0, 47.0)	207	57.0 (38.0, 72.0)	**<0.0001**
Non-traumatic	96	55.5 (42.0, 63.5)	234	63.0 (54.0, 73.0)	**<0.0001**
Severity of injury					
C1-C4, AIS A, B, C	76	34.5 (23.5, 52.0)	59	62.0 (51.0, 71.0)	**<0.0001**
C5-C8, AIS A, B, C	75	33.0 (23.0, 51.0)	32	67.5 (50.5, 77.0)	**<0.0001**
T1-S5, AIS A, B, C	182	35.0 (25.0, 51.0)	169	60.0 (48.0, 73.0)	**<0.0001**
All AIS D	128	43.0 (27.0, 58.0)	181	59.0 ((46.0, 70.0)	**<0.0001**
Bladder management					
IC (AIC + ISC)	289	34.0 (24.0, 48.0)	120	51.0 (34.0, 60.0)	**<0.0001**
IndC (TUC + SPC)	50	61.5 (49.0, 71.0)	231	69.0 (58.0, 77.0)	**0.0012**
Reflex voiding	20	26.0 (21.0, 38.5)	17	40.0 (34.0, 60.0)	**0.0095**
Voluntary voiding	102	40.0 (27.0, 55.0)	73	57.0 (49.0, 63.0)	**<0.0001**

*Wilcoxon rank-sum test (two-sided);

*AIC* intermittent catheterisation by attendant, *ISC* intermittent self-catheterisation, *TUC* transurethral indwelling catheterisation, *SPC* suprapubic catheterisation.

Finally, the proportion of SCI/D patients with IC or indwelling catheter drainage (IndC) of the urinary bladder at the end of primary rehabilitation was examined. Here, too, significant differences in all 12 subcategories without lacking data (missings) were observed (Table 6). The most drastic changes affected tetraplegics. In high tetraplegics, the proportion of IC fell from 61.8 to just 1.7%, and in low tetraplegics from 72.0 to 6.3%. As a consequence, the proportion of IndC increased from 15.8 to 96.6% among high tetraplegics and from 14.7 to 87.5% among low tetraplegics.

**Table 6:** Proportion of patients with intermittent catheterization and permanent catheterization of all patients at discharge from initial rehabilitation in periods 1 and 2.

	1995–1999				2020–2021				p-value
	IC		IndC		IC		IndC		
	N	%	N	%	N	%	N	%	
All	289	62.7	50	10.8	120	27.2	231	52.4	**<0.0001**
Gender									
Male	222	65.5	26	7.7	87	30.0	141	48.6	**<0.0001**
Female	67	54.9	24	19.7	33	21.8	90	59.6	**<0.0001**
Age									
0–15	7	63.6	0	0	0	0	1	100	n/a
16–30	117	71.8	6	3.7	26	53.1	12	24.5	**<0.0001**
31–45	81	67.5	5	4.2	22	41.5	16	30.2	**<0.0001**
46–60	61	59.8	13	11.7	43	35.5	43	35.5	**<0.0001**
61–75	23	41.1	22	39.3	29	21.8	87	65.4	**0.0015**
76+	0	0	4	44.4	0	0	72	85.7	n/a
Spinal cord injury									
Traumatic	245	67.1	32	8.8	54	26.1	108	52.2	**<0.0001**
Non-traumatic	44	45.8	18	18.75	66	28.2	123	52.6	**<0.0001**
Severity									
C1-C4, AIS A, B, C	47	61.8	12	15.8	1	1.7	57	96.6	**<0.0001**
C5-C8, AIS A, B, C	54	72.0	11	14.7	2	6.3	28	87.5	**<0.0001**
T1-S5, AIS A, B, C	147	80.8	20	11.0	67	39.6	88	52.1	**<0.0001**
All AIS D	41	32.0	7	5.5	50	27.6	58	32.0	**<0.0001**

*IC* ISC + AIC, *IndC* TUC + SPC, *IC* intermittent catheterisation, *AIC* assisted intermittent catheterisation, *ISC* intermittent self-catheterisation, *IndC* indwelling catheter, *TUC* transurethral indwelling catheterisation, *SPC* suprapubic indwelling catheterisation.

In a logistic regression analysis (Table 7), female gender, age over 50 years, and all levels of SCI/D except AIS D were significant predictors of bladder management using indwelling catheters (transurethral or suprapubic). Furthermore, increasing age and AIS A-C low- and high-level tetraplegia led to higher odds for indwelling catheter drainage of the urinary bladder.

**Table 7:** Logistic regression results reflecting the dependency of bladder management by transurethral indwelling catheterisation or suprapubic indwelling catheterisation on Gender, Age, Spinal cord injury cause and Severity of injury in both periods.

	Odds ratio	95% CI	p-value
Gender			
Male	Reference category		
Female	1.611	1.083–2.397	**0.0185**
Age			
0–30	Reference category		
31–49	1.481	0.751–2.972	0.2600
50–64	6.120	3.351–11.710	**<0.0001**
65–74	33.385	16.870–69.480	**<0.0001**
75+	80.910	38.197–181.993	**<0.0001**
Spinal cord injury			
Traumatic (TSCI)	Reference category		
Non-traumatic (NTSCI)	1.423	0.929–2.180	0.1044
Severity of injury			
All AIS D	Reference category		
C1-C4, AIS A, B, C	13.553	7.528–24.963	**<0.0001**
C5-C8, AIS A, B, C	6.002	3.112–11.676	**<0.0001**
T1-S5, AIS A, B, C	2.658	1.687–4.245	**<0.0001**

## Discussion

This study investigated demographic, neurological, rehabilitative and neuro-urological changes in SCI patients with traumatic or non-traumatic spinal cord injury over a 25-year period in a tertiary spinal cord injury centre in Germany. To the authors’ best knowledge, no such comprehensive study has been conducted in Germany or other countries to date.

Although there are some studies on the changes in characteristics over time patients with TSCI, similar studies in patients with NTSCI are extremely rare. There are also very few comparative studies on the demographic differences between TSCI and NTSCI patients. Conti et al. [5] reported on the temporal changes in incidence and mortality between 2008 and 2020 in the Italian region of Piedmont for both traumatic and non-traumatic SCI. Lee et al. [3] investigated some epidemiologic changes in patients with TSCI and NTSCI between 1990 and 2019 in South Korea.

Consistently, almost all study groups reported an increase in patient age at onset of SCI/D over the last decades, both in TSCI [3, 8, 10, 11, 14, 15, 23, 24] and in NTSCI [3, 16]. The increase in the number of people affected in the over-60-years of age group is particularly striking and is accompanied by a variety of consequences. This applies to both TSCI [8, 10, 11, 14, 15, 23] and NTSCI [16] and is consistent with the results of our own study population, albeit to a much greater extent for TSCI than for NTSCI. Here, around half of those affected are now 60 years or older at the onset of SCI/D.

With regard to the changes in SCI/D level and injury severity, there are also similarities between our observations and the results of other studies: Several studies described an increase in the proportion of incomplete paraplegics [4, 14, 23], while the proportion of completely paralyzed paraplegics decreased [14, 23]. Nijendijk et al. [11] described an increase in tetraplegics and for incomplete forms of paralysis for a Dutch TSCI population between 1994 and 2016. Only Li et al. [10] described an increase in incomplete paraplegics and a decrease in complete tetraplegics in TSCI for the province of Tianjin in China. In contrast to these data, Choi et al. [16] found a decrease in the frequency of tetraplegics in NTSCI patients in South Korea between 2007 and 2020, and an increase in patients with paraplegia or cauda injury.

Broadly consistent results of the available studies show, in accordance with the results of our own investigations, that the proportion of female patients with SCI/D has increased in recent decades and is significantly higher for NTSCI than for TSCI. While only Lee et al. [4] found no change in the male-female ratio in TSCI patients in South Korea between 1990 and 2019, Li et al. [10] in China, Moschovou et al. [23] in Nordic countries, Nijendijk et al. [11] in the Netherlands and Yokota et al. [14] in Japan consistently found an increasing proportion of female patients with TSCI in recent decades. A direct comparison of the male to female ratio between patients with TSCI and NTSCI showed a ratio of mostly around 80:20 for TSCI and, in contrast, an almost equal sex ratio for NTSCI [3, 6, 18, 25, 26], with broad consistencies in all studies as well as in our own study. Conti et al. [5] also showed a significantly higher proportion of female patients with NTSCI (around 40%) compared to TSCI (just over 20%) with only slight changes in TSCI and an increasing trend in NTSCI over the last 12 years.

Lee et al. [3] found an increase in the frequency of NTSCI in South Korea from 1990–1999 to 2010–2019 from 11.2 to 29.3%. Our own results showed a sharp increase in non-traumatic causes in male patients from 14.7 to 48.3%, and even a reversal in the frequency of causes in female patients: In the period 1995–1999 only approximately a third were non-traumatic, whereas in 2020–2021 approximately two thirds were non-traumatic.

Our data are in line with the well-known fact that patients with NTSCI are on average 10–20 years older at the onset of paralysis than patients with TSCI [3, 5, 6, 18, 26], although according to our data the age difference has tended to decrease in recent decades due to the greater increase in the average age of TSCI.

Although the absolute length of primary rehabilitation after SCI/D differs considerably due to different healthcare systems in the various countries, the primary rehabilitation of patients with NTSCI is significantly shorter than that of patients with TSCI [6, 26, 27, this study] and has become even shorter in recent decades. This applies to patients with TSCI [11] as well as with NTSCI (this study). The specific character of a trauma hospital may play a role here. For example, oncological therapy, which may well be indicated for some NTSCI patients, was not offered in a trauma hospital, possibly leading to early discharge from initial rehabilitation to receive oncologic therapy at another hospital. If hospitals offer oncological therapy in addition to paraplegiological therapy, these patients who have suffered NTSCI as a result of a tumour may stay in hospital longer than patients with TSCI.

What significance do the observed changes over the past decades have on the neuro-urological therapy standards and bladder management? Although patients with older age and NTSCI had higher multimorbidity (higher multimorbidity index MMI [17] or Charlson Score [26]) and a higher proportion with complications during primary rehabilitation [27], the length of primary rehabilitation has decreased for both TSCI and NTSCI. In contrast, it must be assumed that the observed changes of characteristics of SCI/D patients most likely lead to a significantly higher medical treatment effort during primary rehabilitation. Unfortunately, no significant studies have been conducted on this issue to date although there is a considerable need for further studies.

There is also very little data on the destination on discharge from primary rehabilitation following SCI/D. In their somewhat older study, Nijendijk et al. [11] found no significant difference in the destinations on discharge in patients with TSCI between 1994 and 2010. This may be due to the relatively short observation period. In 2024, Lena et al. [6] also reported no significant difference between patients with TSCI or NTSCI in this respect. In contrast, in our own study, there was a significant increase in the proportion of patients discharged to a nursing home between 1995–1999 and 2020–2021, while the number of those who could be discharged to a disabled-friendly converted home fell significantly. This must certainly also be seen as a consequence of the higher average age at the onset of SCI/D in the second period. However, country- and insurance-specific factors must also be taken into account in this discussion.

It is known from several studies from the Netherlands and Switzerland that a cervical level of SCI/D and older age [28] or an age over 60 years [29] or 65 years [30] act as negative predictors for bladder management with indwelling suprapubic catheterisation in logistic regression analyses. And, of course, the changed demographic and clinical characteristics of SCI/D patients at the time of their primary rehabilitation observed in the last decades also have an impact on bladder management at the end of primary rehabilitation and will enhance this trend. These changes lead to a considerable proportion of initial rehabilitation SCI/D patients not being able to apply the standards of bladder management, e.g., emptying the bladder by IC. Our own findings confirm this for SCI/D patients at the end of initial rehabilitation who are female, older, and have a higher severity of SCI/D.

Although IC remains the gold standard for bladder emptying after SCI/D, when recommending adequate bladder management, aspects such as independence from external assistance and the quality of life of those affected, or possible side effects of the anticholinergic drugs often required for IC to ensure continence during the storage phase, especially in older patients, must of course also be taken into account.

Although attempts should always be made to establish the gold standard IC, the following limitations of IC must be acknowledged [31]: (1) limited motor function of the upper extremities; (2) anatomical limitations (female or obese); and (3) limited functional bladder capacity (reduced compliance or detrusor overactivity).

Overall, patient-reported outcomes have rarely been measured to date [32], but a study of nearly 1500 SCI/D patients [33] showed that bladder drainage via an indwelling catheter was associated with fewer lower urinary tract symptoms than IC in both paraplegic and tetraplegic patients.

The Canadian guidelines for the diagnosis, management, and surveillance of neurogenic lower urinary tract dysfunction recommend that the selection of an assisted bladder drainage method (IC, urethral, or suprapubic catheter) should be individualized to the patient’s motor functions, anatomical limitations, bladder characteristics, prior urological complications, and Qol [34].

However, a recent systematic review of evidence-based recommendations for the rehabilitation and management of the aging SCI/D population revealed a notable lack of high-quality aging-related recommendations in current clinical practice guidelines [35].

The question of optimal individual bladder management must therefore also be discussed against the background of the changing demographic structure of patients undergoing initial rehabilitation for SCI/D. When selecting the optimal individual method of bladder emptying, a multidimensional approach is increasingly necessary. The risks and benefits of different bladder emptying methods must be carefully weighed up.

Although the present study clearly enhances the data base in this field, the study also has several limitations. The retrospective data collection and the single-centre design may bias the results. This study is driven by the way the German healthcare system is organized. Thus, comparisons with results from other countries or regions may be difficult. This applies to various aspects of the entire initial rehabilitation process following SCI/D, from the duration of inpatient rehabilitation to discharge management options and bladder management. Furthermore, reimbursement for disposable catheters is also regulated very differently in various countries.

## Summary

SCI/D patients today are significantly older at the onset of paralysis, more often female, more often paralyzed due to a non-traumatic cause and more often incompletely paralyzed than 25 years ago. Compared to TSCI, patients with NTSCI are on average significantly older at the onset of paralysis, more often female and more often have incomplete paralysis. Nevertheless, the duration of primary rehabilitation has decreased for both TSCI and NTSCI patients. Bladder management on discharge from primary rehabilitation has also changed significantly: The proportion of patients with intermittent catheterisation has decreased dramatically, while the proportion of permanent catheter patients has increased significantly.

## Data Availability

The datasets generated during and/or analysed during the current study are available from the corresponding author on reasonable request.

## References

[1] Mitton C, Dionne F, Fallah N, Noonan VK. An economic analysis of the association among secondary health conditions, health care costs, and quality of life for persons with spinal cord injury. Top Spinal Cord Inj Rehabil. 2023;29:80–88. 38076292 10.46292/sci22-00039 PMC10644854

[2] Diop M, Epstein D. A systematic review of the impact of spinal cord injury on costs and health-related quality of life. PharmacoEconomics - Open. 2024;8:793–808. 39150624 10.1007/s41669-024-00517-3 PMC11499558

[3] Lee B-S, Kim O, Ham D. Epidemiologic changes in nontraumatic spinal cord injury for the last 30 years (1990–2019) in South Korea. Spinal Cord. 2022;60:268–73. 34453110 10.1038/s41393-021-00695-5

[4] Lee B-S, Kim O, Ham D. Epidemiological changes in traumatic spinal cord injuries for the last 30 years (1990–2019) in South Korea. Spinal Cord. 2022;60:612–7. 34465888 10.1038/s41393-021-00694-6

[5] Conti A, Campagna S, Gianino MM, Mamo C, Onorati R, Albanesi B, et al. Incidence and mortality of spinal cord injury from 2008 to 2020: a retrospective population-based cohort study in the Piedmont Region, Italy. Spinal Cord. 2023;61:99–105. 35933474 10.1038/s41393-022-00842-6 PMC9362101

[6] Lena E, Timelli L, Di Fonzo S, Tonini A, Pisani V, Garcovich C, et al. Unveiling the mosaic: comparing demographics and outcomes in traumatic vs. non-traumatic spinal cord injuries. Eur J Phys Rehabil Med. 2024;60:980–8. 39352291 10.23736/S1973-9087.24.08554-X PMC11729709

[7] Ding W, Hu S, Wang P, Kang H, Peng R, Dong Y, et al. Spinal Cord Injury: the global incidence, prevalence, and disability from the global burden of disease study 2019. Spine. 2022;47:1532–40. 35857624 10.1097/BRS.0000000000004417 PMC9554757

[8] Hao D, Du J, Yan L, He B, Qi X, Yu S, et al. Trends of epidemiological characteristics of traumatic spinal cord injury in China, 2009–2018. Eur Spine J. 2021;30:3115–27. 34392419 10.1007/s00586-021-06957-3

[9] Hu Y, Li L, Hong B, Xie Y, Li T, Feng C, et al. Epidemiological features of traumatic spinal cord injury in China: a systematic review and meta-analysis. Front Neurol. 2023;14:1131791. 37021283 10.3389/fneur.2023.1131791 PMC10069652

[10] Li H-L, Xu H, Li Y-L, Sun S-W, Song W-Y, Wu Q, et al. Epidemiology of traumatic spinal cord injury in Tianjin, China: an 18-year retrospective study of 735 cases. J Spinal Cord Med. 2019;42:778–85. 29323634 10.1080/10790268.2017.1415418 PMC6830263

[11] Nijendijk JH, Post MW, Van Asbeck FW. Epidemiology of traumatic spinal cord injuries in the Netherlands in 2010. Spinal Cord. 2014;52:258–63. 24445971 10.1038/sc.2013.180

[12] Chiu AK, Pease TJ, Prakash H, Oster BA, Smith RA, Sahlani M, et al. The changing epidemiology of traumatic spine injuries: a trends analysis of 26 years of patients at a major level 1 trauma center in the United States. Spine J. 2024;24:1561–70. 38843959 10.1016/j.spinee.2024.05.009

[13] Sun J, Yuan W, Zheng R, Zhang C, Guan B, Ding J, et al. Traumatic spinal injury-related hospitalizations in the United States, 2016–2019: a retrospective study. Int J Surg. 2023;109:3827–35. 37678281 10.1097/JS9.0000000000000696 PMC10720809

[14] Yokota K, Sakai H, Kawano O, Morishita Y, Masuda M, Hayashi T, et al. Changing trends in traumatic spinal cord injury in an aging society: epidemiology of 1152 cases over 15 years from a single center in Japan. PLOS ONE. 2024;19:e0298836. 38753862 10.1371/journal.pone.0298836 PMC11098516

[15] Thorogood NP, Noonan VK, Chen X, Fallah N, Humphreys S, Dea N, et al. Incidence and prevalence of traumatic spinal cord injury in Canada using health administrative data. Front Neurol. 2023;14:1201025. 37554392 10.3389/fneur.2023.1201025 PMC10406385

[16] Choi Y, Leigh J-H, Jeon J, Lee GJ, Shin H-I, Bang MS. Trends in the incidence and etiology of non-traumatic spinal cord injury in Korea: a nationwide population-based study from 2007 to 2020. J Korean Med Sci. 2023;38:e158. 37158777 10.3346/jkms.2023.38.e158 PMC10166702

[17] Hong HA, Fallah N, Wang D, Cheng CL, Humphreys S, Parsons J, et al. Multimorbidity in persons with non-traumatic spinal cord injury and its impact on healthcare utilization and health outcomes. Spinal Cord. 2023;61:483–91. 37604933 10.1038/s41393-023-00915-0

[18] Molinares DM, Gater DR, Daniel S, Pontee NL. Nontraumatic spinal cord injury: epidemiology, etiology and management. J Pers Med. 2022;12:1872. 36579590 10.3390/jpm12111872 PMC9694799

[19] Rau Y, Schulz AP, Thietje R, Matrisch L, Frese J, Hirschfeld S. Incidence of spinal cord injuries in Germany. Eur Spine J. 2023;32:601–7. 36371751 10.1007/s00586-022-07451-0 PMC9660155

[20] Kirshblum S, Snider B, Rupp R, Read MS. Updates of the international standards for neurologic classification of spinal cord injury. Phys Med Rehabil Clin N Am. 2020;31:319–30. 32624097 10.1016/j.pmr.2020.03.005

[21] Biering-Sørensen F, DeVivo MJ, Charlifue S, Chen Y, New PW, Noonan V, et al. In-ternational Spinal Cord Injury Core Data Set (version 2.0) - including standardization of reporting. Spinal Cord. 2017;55:759–64. 28555665 10.1038/sc.2017.59

[22] DeVivo MJ, Biering-Sørensen F, New P, Chen Y. Standardization of data analysis and reporting of results from the International Spinal Cord Injury Core Data Set. Spinal Cord. 2011;49:596–9. 21135863 10.1038/sc.2010.172

[23] Moschovou M, Antepohl W, Halvorsen A, Pettersen AL, Divanoglou A. Temporal changes in demographic and injury characteristics of traumatic spinal cord injuries in Nordic countries - a systematic review with meta-analysis. Spinal Cord. 2022;60:765–73. 35220414 10.1038/s41393-022-00772-3

[24] Wang C, Shang S, Hou M, Wang J, Kang Y, Lou Y, et al. Epidemiological age-based differences in traumatic spinal cord injury patients: a multicenter study based on 13,334 inpatients. J Spinal Cord Med. 2025;48:232–40. 38426946 10.1080/10790268.2024.2309716 PMC11864031

[25] Scivoletto G, Farchi S, Laurenza L, Molinari M. Traumatic and non-traumatic spinal cord lesions: an Italian comparison of neurological and functional outcomes. Spinal Cord. 2011;49:391–6. 20603629 10.1038/sc.2010.85

[26] Guilcher SJT, Munce SEP, Couris CM, Fung K, Craven BC, Verrier M, et al. Health care utilization in non-traumatic and traumatic spinal cord injury: a population-based study. Spinal Cord. 2010;48:45–50. 19546877 10.1038/sc.2009.78

[27] Josefson C, Rekand T, Lundgren-Nilsson Å, Sunnerhagen KS. Epidemiology of spinal cord injury in adults in Sweden, 2016–2020: a retrospective registry-based study. Neuroepidemiology. 2025;59:334–42. 39137737 10.1159/000540818 PMC12324699

[28] Poublon CG, Scholten EWM, Wyndaele MIA, Post MWM, Stolwijk-Swüste JM. Changes in bladder emptying during inpatient rehabilitation after spinal cord injury and predicting factors: data from the Dutch Spinal Cord Injury Database. Spinal Cord. 2023;61:624–31. 37608226 10.1038/s41393-023-00925-y

[29] Anderson CE, Birkhäuser V, Liechti MD, Jordan X, Luca E, Möhr S, et al. Sex differences in urological management during spinal cord injury rehabilitation: results from a prospective multicenter longitudinal cohort study. Spinal Cord. 2023;61:43–50. 36224336 10.1038/s41393-022-00860-4 PMC7614058

[30] Krebs J, Wöllner J, Rademacher F, Pannek J. Bladder management in individuals with spinal cord injury or disease during and after primary rehabilitation: a retrospective cohort study. World J Urol. 2022;40:1737–42. 35599284 10.1007/s00345-022-04027-x

[31] Zlatev DV, Shem K, Elliott CS. How many spinal cord injury patients can catheterize their own bladder? The epidemiology of upper extremity function as it affects bladder management. Spinal Cord. 2016;54:287–91. 26782841 10.1038/sc.2015.169

[32] Sorokin I, De E. Options for independent bladder management in patients with spinal cord injury and hand function prohibiting intermittent catheterization. Neurourol Urodyn. 2015;34:167–76. 24151101 10.1002/nau.22516

[33] Myers JB, Lenherr SM, Stoffel JT, Elliott SP, Presson AP, Zhang C, et al. Patient reported bladder related symptoms and quality of life after spinal cord injury with different bladder management strategies. J Urol. 2019;202:574. 30958741 10.1097/JU.0000000000000270

[34] Kavanagh A, Baverstock R, Campeau L, Carlson K, Cox A, Hickling D, et al. Canadian Urological Association guideline: diagnosis, management, and surveillance of neurogenic lower urinary tract dysfunction - full text. Can Urol Assoc J. 2019;13:E157–E176. 30763235 10.5489/cuaj.5912 PMC6570608

[35] Seijas V, Schrepfer L, Posada AM, Spir MA, Machado B, Sigrist-Nix D, et al. Evidence-based recommendations for the rehabilitation and management of the ageing population with spinal cord injury: a systematic review of clinical practice guidelines. Eur J Phys Rehabil Med. 2024;60:433–44. 38551520 10.23736/S1973-9087.24.08244-3 PMC11255876

